# A free association semantic task for fNIRS-based perinatal depression assessment

**DOI:** 10.3389/fneur.2024.1491923

**Published:** 2025-01-15

**Authors:** Danni Chen, Xuanjin Yang, Yuanyuan Liang, Chen Huang, Suhan Zhang, Yini Li, Ye Li, Xiaofei Li, Wenting Mu, Dan Zhang, Liangkun Ma

**Affiliations:** ^1^Department of Psychological and Cognitive Sciences, Tsinghua University, Beijing, China; ^2^National Clinical Research Center for Obstetric & Gynecologic Diseases Department of Obstetrics and Gynecology, Peking Union Medical College Hospital, Chinese Academy of Medical Sciences and Peking Union Medical College, Beijing, China; ^3^School of Humanities and Social Sciences, Chinese Academy of Medical Sciences and Peking Union Medical College, Beijing, China

**Keywords:** perinatal depression, functional near-infrared spectroscopy, free association semantic task, verbal fluency test, clinical assessment

## Abstract

Perinatal depression (PD) is a highly prevalent psychological disorder that has a detrimental effect on infant and maternal physical and mental health, but effective and objective assessment of PD is still insufficient. In recent years, the functional near-infrared spectroscopy (fNIRS) has been acknowledged as an effective non-invasive tool for clinical assessment of depression. This study proposed a free association semantic task (FAST) paradigm for fNIRS-based assessment of PD. To better address the emotion characteristics of PD, the participants are required to generate a dynamic concept chain based on positive, negative or neutral seed words, while 48-channel fNIRS recordings over frontal and bilateral temporal regions. Results from twenty-two late-pregnant women revealed that, the oxyhemoglobin (oxy-Hb) changes during the FAST with the positive and negative seed words over the frontal region were correlated with PD severity, which was different from the correlation patterns in the FAST with neutral seed word and the classical verbal fluency test (VFT). Furthermore, distinct correlation patterns were also observed in the FAST with the positive and negative seed words, manifested in fNIRS channels corresponding to the right dorsolateral prefrontal cortex (DLPFC) and right inferior frontal gyrus (IFG), respectively. Moreover, regression analyses showed that the FAST with positive and negative seed words can well explain the severity of PD. Our findings suggest the proposed FAST paradigm as a promising approach for PD assessment.

## 1 Introduction

Perinatal depression (PD) is defined as a major depressive episode that occurs from the time of pregnancy until four 4 weeks after delivery (1). It is estimated that the prevalence of PD ranges from 11 to 20% (2–4), with a rate of about 16.3% in China (5). PD has detrimental effects on both physical and mental health, leading to an increased risk of pre-eclampsia, gestational diabetes, and preterm birth, as well as low mood, loss of interest, insomnia (6–8). It could also have a critical influence on the development of the newborns, potentially increasing their risk of having cognitive, emotional and behavioral issues (9–12). As the gold standard for perinatal mental health screening (13), the Edinburgh Postnatal Depression Scale (EPDS) has been widely recommended and used in clinical settings for its validity, brevity, and user-friendly features (14). The use of the EPDS in perinatal populations has been instrumental in identifying individuals who may require further assessment for depression and subsequent intervention (14, 15).

With the development of cognitive neuroscience over the past decade, biomarkers and pathological mechanisms of depression have been recognized as the key to improving diagnostic accuracy and treatment effectiveness (16, 17). Functional Near Infrared Spectroscopy (fNIRS) could be a promising tool for assessing perinatal depression. fNIRS utilizes the scattering of 650–1,000 nm near-infrared light by the principal components of blood to quantify alterations in cerebral hemoglobin concentration via the neurovascular coupling mechanism (18, 19). Compared to other neuroimaging techniques such as electroencephalography (EEG) and functional magnetic resonance imaging (fMRI), fNIRS offers several advantages, including low operating cost, insensitivity to the subject’s body movement related noise and environmental electromagnetic noise, high portability and thus high deployment flexibility, etc. (20, 21). These benefits make it particularly suitable for use in challenging clinical settings such as routine outpatient examinations for pregnant women (20). Although the use of fNIRS for PD assessment is still limited, it has been widely applied for the assessment of depression in the general population (22–24).

The verbal fluency test (VFT) is currently the most widely used paradigm for fNIRS-based depression assessment. The participants are instructed to verbally generate as many words as possible within a specified category (e.g., a phoneme or a word) in a given time period (25–27). The depressed people have been shown to generate fewer words and have a relatively smaller increase in oxyhemoglobin (oxy-Hb) in the frontal and temporal regions compared to healthy controls (HCs) (24, 28–31). More importantly, correlation analysis also indicated that the VFT-based fNIRS activities over the fNIRS channels corresponding to the dorsolateral prefrontal cortex (DLPFC), frontopolar cortex (FPC), inferior frontal gyrus (IFG), temporopolar area, and pre-motor area, have been linked to the severity of depression (22, 32–36). The VFT is known to assess depression primarily by measuring individuals’ cognitive abilities and executive functioning (29, 37). However, given that depression is an affective disorder characterized by core symptoms such as low mood, feelings of worthlessness, and rumination (1), the VFT may not comprehensively capture the essence of depression. This limitation could potentially reduce its effectiveness in targeted clinical applications.

The recently developed Free Association Semantic Task (FAST) paradigm could be a suitable candidate to extend the VFT paradigm, for an enhanced specificity for depression assessment. The structure of the FAST paradigm is similar to that of the VFT, requiring participants to generate a dynamic concept chain following a given seed word. However, unlike the VFT, the seed words of FAST could be emotion-related, and its paradigm requires participants to produce the next word based on the immediately preceding word in the sequence, rather than always revolving around the seed word. This design is advantageous for better capturing the dynamic process of cognitive arise, unfold and transition over time and facilitating the exploration of the underlying neural mechanisms of word generation and retrieval (38, 39). The potential utility of FAST in characterizing depression is underscored by recent findings that highlight its sensitivity to ruminative tendencies: using emotion-related words as seed words, individuals prone to high levels of rumination exhibit a propensity to shift from positive to negative associations, a behavior that is indicative of depressive symptomatology (38). Furthermore, chains of negative associations, as elicited by the FAST paradigm, have been identified as robust predictors of repetitive negative thinking and the manifestation of negative emotional traits (40). In the meanwhile, an fMRI study has identified specific brain patterns linked to the emotional valences of word chains generated by the participants, including the dorsomedial prefrontal cortex (DMPFC), ventromedial prefrontal cortex (VMPFC), orbitofrontal cortex (OFC), and hippocampus (39). In these studies, emotional words have been used as the seed word to elicit the word chain and its effectiveness for reflecting depression has been demonstrated. Nevertheless, to our knowledge, the FAST paradigm has not been used for clinical samples and no fNIRS studies have been reported.

The present study aimed to investigate the feasibility of the Free Association Semantic Task paradigm for fNIRS-based perinatal depression assessment. fNIRS recordings over the frontal and temporal regions were employed to have a comprehensive coverage over the related brain areas (29, 41). In order to better target the characteristics of depression, three emotional seed words of positive, negative or neutral valences were selected as seed words (38, 39). Considering the context of possible clinical application (31, 35), correlation and regression analyses for quantitatively assessing the severity of perinatal depression were conducted, rather than classifying individuals as depressed or not depressed. Late-pregnant women were recruited for the present study, for the following reasons: (1) the late-pregnant period is a time when expectant mothers may begin to experience increased depressive symptoms due to the anticipation of childbirth and the changes that come with motherhood (42, 43); and (2) identifying depression during the late-pregnant period allows for early intervention and support that would potentially reduce the risk of postpartum depression (44). Meanwhile, the classical VFT paradigm was employed as a comparison condition to explore the potential unique features and benefits of FAST. Referring to related studies on fNIRS for depression in general (22–24) and the fMRI study with FAST paradigm (39), we hypothesize that the fNIRS activities during FAST execution would reflect the severity of PD and the involved brain regions might be more focused over the prefrontal regions as compared to VFT. Moreover, the FAST-related brain regions may differ based on the valence of the seed words (45, 46) and it would be practically important to know which seed word(s) were more effective. The findings of this study are expected to deepen the understanding of the neural mechanisms of PD, and contribute to the implementation of the clinical quantitative assessment of PD.

## 2 Methods

### 2.1 Participants

A total of 24 women in late pregnancy (after 28 weeks of gestation) were recruited for this study. The age range was between 24 and 42 years (mean age ± standard deviation [SD]: 33.29 ± 4.40 years). Participants were excluded if they had any of the following conditions: (1) current major physical illness or other mental disorder, (2) history of alcohol or drug abuse, or (3) multiple gestations. Two of the participants only completed the VFT and did not complete the FAST due to scheduling conflicts. The study was approved by the Ethics Committee of Peking Union Medical College Hospital and written informed consent was obtained from all participants (Ethics number: I-22PJ122).

### 2.2 fNIRS measurement

In this study, a 48-channel NirSmart-6000A device (Danyang Huichuang Medical Equipment Co., Ltd., China) was employed to continuously measure and record cerebral blood flow in brain oxygenated hemoglobin (Oxy-Hb) and deoxyhemoglobin (HbR) concentrations at two wavelengths (730 nm and 850 nm) in the near-infrared light. The system employs a light-emitting diode (LED) as the near-infrared light source and an avalanche photodiode (APD) as the detector, with the source-detector distance of 3 cm. The sampling rate was set at 11 Hz.

**Figure 1:**
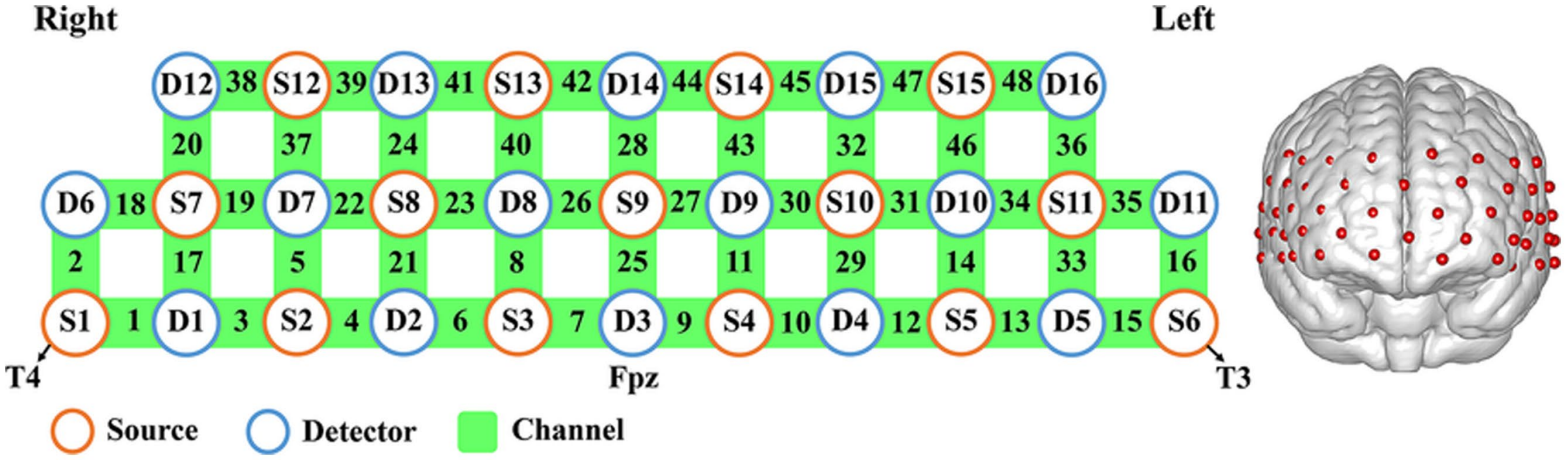
Description of fNIRS channel location. Relationship between source (S)–detector (D) and corresponding channel location map.

This study utilized a total of 15 light sources and 16 detectors, covering frontal and bilateral temporal regions (Figure 1). The detector 3 (D3) was set at Fpz, and the source 1 and 6 (S1 and S6) were placed as T4 and T3, according to the international 10–20 system. For the probabilistic registration of all fNIRS channels onto the cortical surface, the topography data of cranial landmarks were projected into a 3D reference frame (MNI-space, Montreal Neurological Institute) (47, 48) based on NIRS_SPM (49). Spatial registration information could thus be provided for each channel, with standard deviations ranging from 4.7 to 7.0 mm (48). The relevant anatomical labels and the percentage of overlap for each channel were presented in Supplementary Table 1.

### 2.3 Procedure

**Figure 2:**
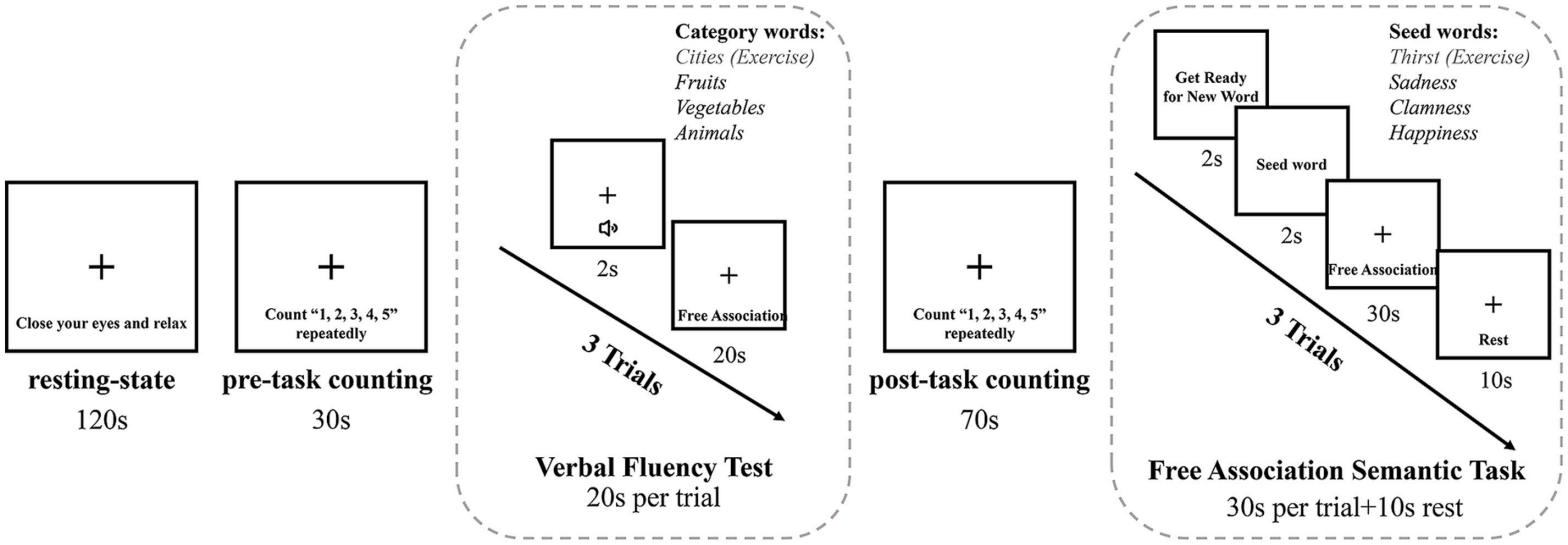
Experimental flow diagram.

The participants participated in the study when they paid their regular visit to the hospital for their pregnancy check-ups. During the study, participants were required to maintain a comfortable seated position in order to minimize possible body and head movement. Upon task initiation, the participants first engaged in a resting-state phase. They were asked to close their eyes, relax their bodies, remain as still as possible, and try not to think about anything in particular for 2 min. This allowed them to settle into the task state and relax. After that, a pre-task counting stage was followed, serving as a baseline for the subsequent verbal task. During this phase, participants were simply instructed to count the number “1, 2, 3, 4, 5” repeatedly for 30-s. Following this, three consecutive trials of VFT were executed, with a 70-s post-task counting stage immediately ensuing to return blood oxygen levels to the baseline. Given the potential confounding factors induced by emotional tasks, the FAST was performed at the end of the procedure in three trials, with each trial followed by a rest period to ensure recovery of blood oxygen levels to baseline. See the subsequent sections and Figure 2 for more details of VFT and FAST.

After completing the entire task procedure, the Edinburgh Postnatal Depression Scale (EPDS) was employed to assess participants’ level of depression. The scale is a clinically validated and widely utilized self-report questionnaire comprising 10 items, with a score range of 0–30. A higher score indicates a more severe depressive tendency (50, 51). A score of 10 was used as the cut-off point for identifying the presence of depressive tendencies, which has been shown to have good sensitivity and specificity in late pregnancy (52). In addition, basic demographic information was also collected for each participant, including age, gestational weeks, ethnicity, education level, annual household income, sleep quality, and both their own and family’s medical history.

#### 2.3.1 Verbal fluency test

During the classical VFT phase, participants were required to generate as many words as possible that belonged to the corresponding category, beginning with fruits, vegetables, and animals in sequence. The three trials were performed consecutively for 20-s each, for a total task duration of 60-s. To ensure that participants were clear about the task requirements, they were asked to complete a 20-s exercise in the “city” category ahead of the formal task. The number of words generated by the subject in each category, excluding repeated words, was used to quantify task performance (26, 31, 53).

#### 2.3.2 Free association semantic task

Before the start of each trial, there was a 2-s preparation period in which participants were prompted to prepare for the seed word. Immediately afterwards, a “seed word” was presented in black font in the center of a white screen for 2-s, and then for the following 30-s, the participants were asked to generate a dynamic chain of concepts containing a series of words based on the seed word. A 10-s rest period was implemented between trials to allow oxygen to return to baseline levels. The task comprised three trials, with the seed words being “sadness” (negative valence), “calmness” (neutral valence), and “happiness” (positive valence). The neutral seed word trial was positioned in the middle to act as a buffer between the negative and positive seed word trials. Furthermore, to eliminate order effects, the negative and positive seed word trials were counterbalanced between subjects. Prior to the formal task, the non-emotional seed word “thirst” was employed in a practice phase to guarantee that participants had a comprehensive understanding of the task requirements. It is notable that the task required participants to generate concepts each time that were related only to the preceding word in the chain, rather than the seed words. For example, in a trial with the seed word “sadness,” participants may form a word chain such as the string: sadness-crying-painful-destruction-rebirth-peaceful. Thus, the participants’ dynamic thought processes could be quantified in terms of the unfolding of the word chain (38).

All of the above task programs were designed using E-prime 3.0 (Psychology Software Tools Inc., United States).

### 2.4 fNIRS data processing

First, channels were trimmed based on signal strength and standard deviation using the “hmrR_PruneChannels” function (SNRthresh = 2; SDrange = [0, 45]). Then, the raw light intensity was converted to optical density for subsequent analysis. For baseline shifts and spikes in the signal, spline interpolation and Savitzky–Golay filtering were used for correction, respectively (54). And the bandpass filter was set to 0.01–0.5 Hz to remove noise, such as heartbeat and breathing. After that, the data were segmented into trials and corrected for each trial using the last 5 s of the pre-task phase as the baseline. Finally, the optical density data were converted to delta hemoglobin concentration (DC) for later statistical analysis. During the pre-processing, the PHOEBE algorithm (55) and the correlation between Oxy-Hb and HbR was employed to eliminate channels with extreme values. Additionally, the maximum proportion of saturating and flooring points was set at >10% to effectively exclude channels of poor quality (41). The retention rate after data scrubbing was 97.6%. In subsequent analyses, only changes in Oxy-Hb were statistically analyzed, as it has been demonstrated to be more sensitive than HbR concentration in reflecting task-induced changes in local cerebral blood flow (31, 56).

The Homer3 and FieldTrip toolboxes of Matlab R2023b (MathWorks Inc., United States) were used to process the fNIRS data.

### 2.5 Statistical analysis

For the behavioral data, the number of words generated in the two tasks was calculated. Subsequently, the word vectors of category words in VFT, seed words in FAST, and each word generated by participants were calculated using the HanLP package (57). Then, the cosine distance between each category/seed word vector and every word vector in each chain was calculated separately and took 5-s as the unit for average. Through this, an analysis was conducted comparing the distance dynamic trend based on the matching and non-matching of seed words and word chains. Additionally, in the VFT, the classification accuracy for each category was assessed by comparing the distance of each word to the three category words. The category with the minimum distance was identified as the correct classification.

**Figure 3:**
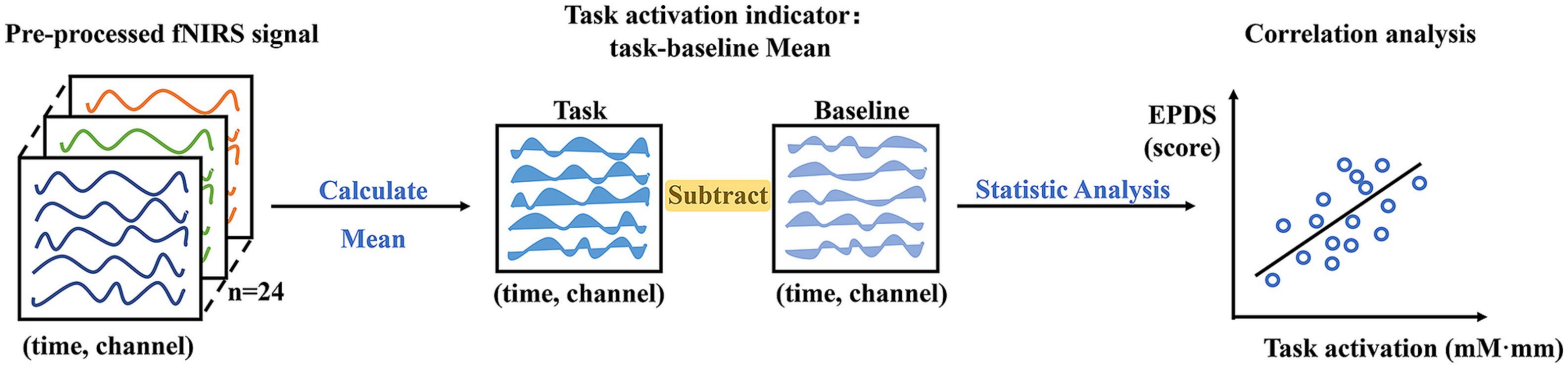
FNIRS statistical analysis flowchart. Note: mM·mm represents “m mol/L * mm” of hemoglobin concentration.

For the pre-processed fNIRS data, the continuous oxy-Hb change of each channel under each task and participant was averaged. And the task activation indicator was defined as the mean oxy-Hb value of the task minus the baseline (pre-task counting period) in order to eliminate the potential for interference from the pure speech process. Given our primary interest in the quantitative assessment of depression, the Pearson correlation analysis was employed to calculate the fNIRS activities during FAST execution in relation to the EPDS (Figure 3). This allowed us to examine the differential characteristics of brain representations during tasks with different emotional seed word. A high correlation value would indicate that the channel has a more sensitive oxy-Hb response as depression levels changes.

Based on this, the same method was used to compare and analyze the fNIRS activities during VFT execution. Meanwhile, to explore whether there were differences between the two tasks in the quantitative assessment of depression, the significant channels under different tasks were extracted for correlation analysis.

Overall, in order to evaluate the explanatory power of each task on depression severity, the significant channels of correlation analysis under each task were selected and a linear regression analysis was performed. A greater determination coefficient (R^2^) would reflect a heightened explanatory validity of the model for depression severity.

For all of the above analyses, a level of significance of *p* < 0.05 was set. The permutation test (5,000 iterations) was used to validate the reliability of the data, followed by correction for multiple comparisons using the false discovery rate (FDR, *p* < 0.05) (58).

Behavioral data were analyzed using Python 3.11.9 (Python Software Foundation, United States), the fNIRS data were conducted in Matlab R2023b (MathWorks Inc., United States) and visualized using the EasyTopo toolbox (59).

## 3 Results

### 3.1 Participant demographic and behavioral outcomes

According to the EPDS score, with a cut-off point of 10 (52), seven participants could be classified as depressed and the other 17 as mentally healthy, suggesting a good diversity of the participants. Neither the participants nor their families reported the presence of other serious mental disorders.

**Figure 4:**
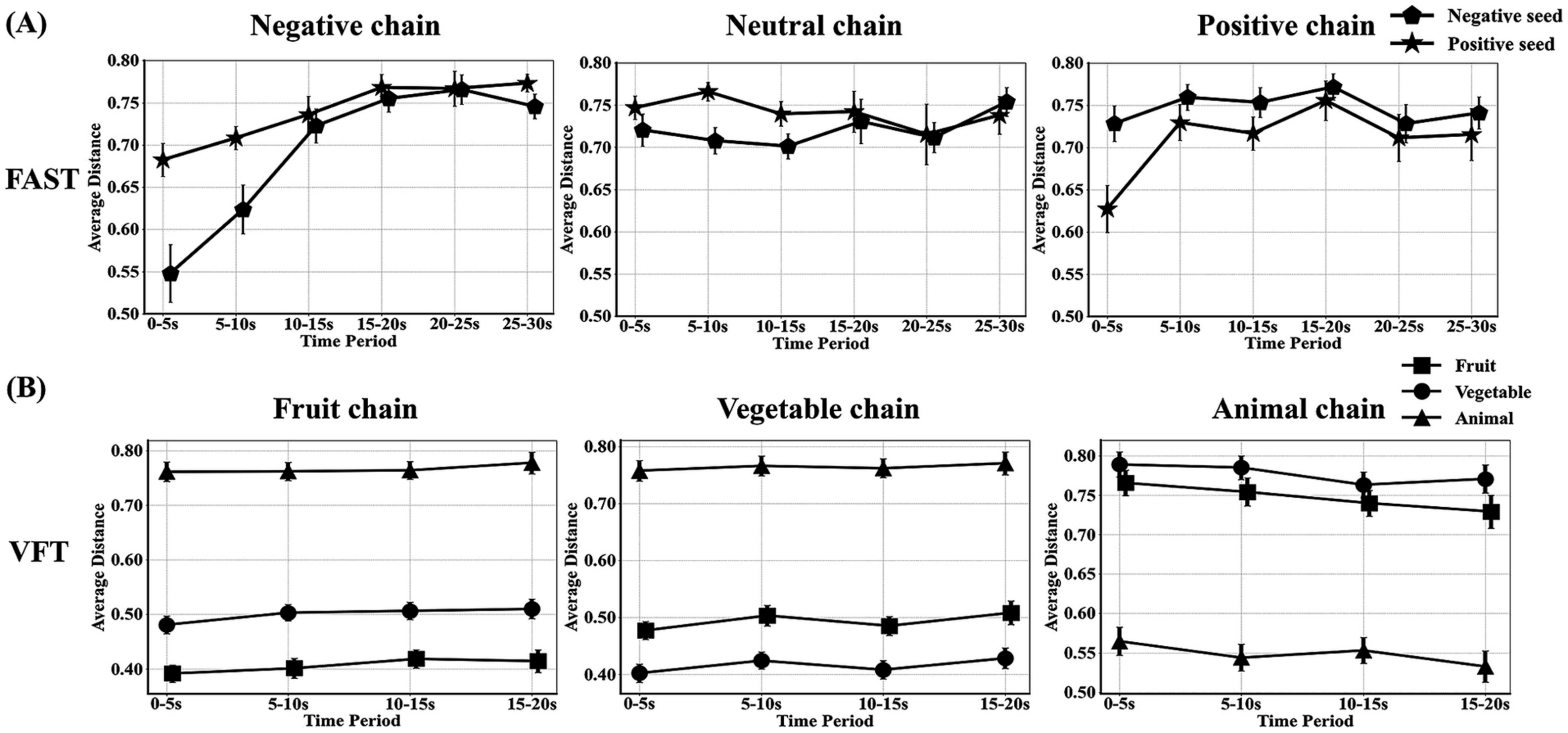
Dynamic analysis of word chains in FAST and VFT. **(A,B)** represent trend graphs of the cosine distance between the seed word and generated words by each subject over time in the FAST and VFT, respectively (averaged in windows of 5-s).

In word chain analysis, the FAST revealed a gradual increase in the dynamic tendency of cosine distances between matched positive and negative seed words and word chains. In contrast, the distance between mismatched items displayed no such trend, exhibiting a stable range of 0.7–0.8 (Figure 4A). While in the VFT task, a distinction can be observed in the cosine distance between matching and unmatching items, with the matching conditions consistently maintained at a relatively minimal distance (Figure 4B). Classification results showed that the accuracy was 98.34% (fruit), 100.00% (vegetable) and 96.53% (animal).

### 3.2 FAST response associated with EPDS

**Figure 5:**
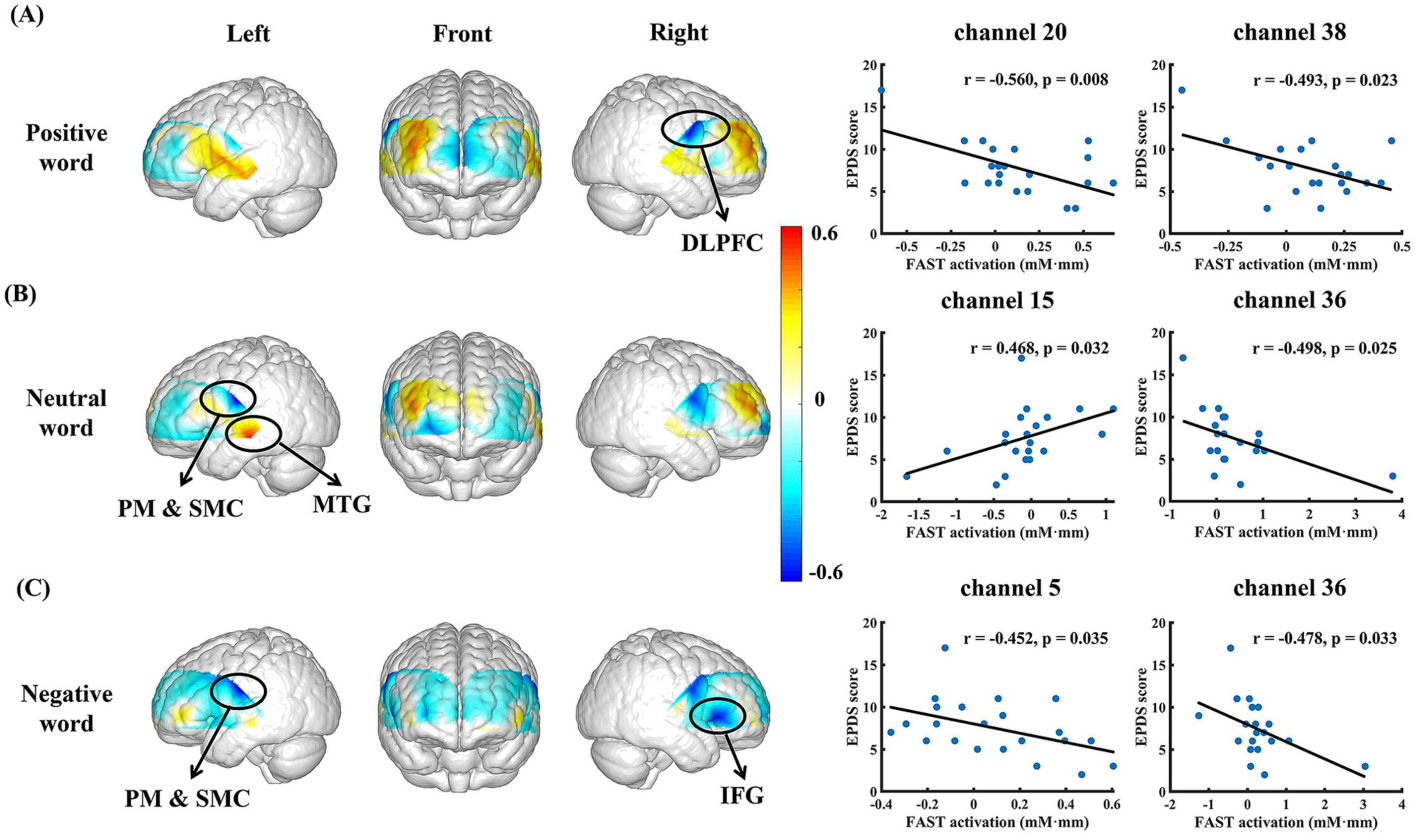
Significant responses of depression-related brain regions in FAST. **(A–C)** represent brain regions and scatter plots corresponding to significant depression-related fNIRS channels in the FAST with positive, neutral, and negative seed words, respectively.

In the FAST with positive seed word, significant negative correlations were observed between EPDS and the mean oxy-Hb levels in channels 38 (dorsolateral prefrontal cortex, DLPFC) and 20 (pre-motor and supplementary motor cortex, PM & SMC; ch 38, *r* = −0.493, *p* = 0.023; ch 20, r = −0.560, *p* = 0.008). As for the negative seed word FAST, the mean Oxy-Hb levels in channels 5 (inferior frontal gyrus, IFG) and 36 (PM & SMC) also showed a significant negative correlation with the EPDS (ch 5, *r* = −0.452, *p* = 0.035; ch 36, *r* = −0.478, *p* = 0.033; Figures 5A,C).

Besides, the EPDS was correlated with mean Oxy-Hb levels in channels 15 (middle temporal gyrus, MTG) and 36 (PM & SMC) during the neutral seed word FAST (ch 15, *r* = 0.468, *p* = 0.032; ch 36, r = −0.498, *p* = 0.025; Figure 5B).

### 3.3 Comparison between FAST and VFT

**Figure 6:**
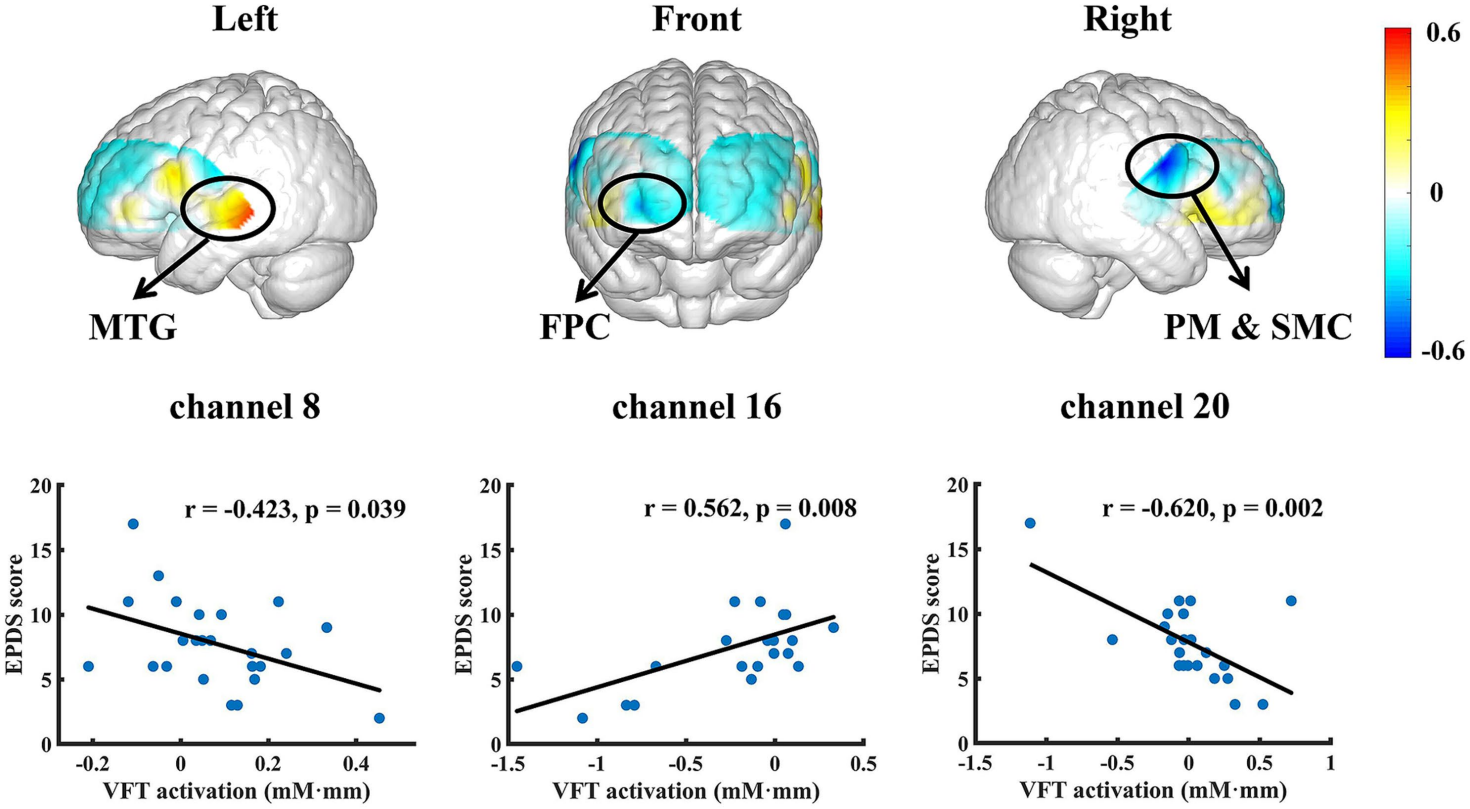
Significant responses of depression-related brain regions in VFT.

Compared to FAST, VFT has notable similarities with neutral seed word FAST. The mean Oxy-Hb levels in channels 15, 16 (MTG) and 20 (PM & SMC) correlated significantly with EPDS (ch 15, *r* = 0.464, *p* = 0.026; ch 16, *r* = 0.562, *p* = 0.008, ch 20, *r* = −0.620, *p* = 0.002). Moreover, there was also a significant negative correlation between EPDS and the mean Oxy-Hb level in channel 8 (frontopolar area, FPC; *r* = −0.423, *p* = 0.039; Figure 6).

To identify possible distinct patterns of depression-related oxy-Hb activation elicited by different tasks, the degree of correlation between the significant channels of each task were calculated. The results indicated the correlations between task-specific channels were mostly insignificant, suggesting that each task exhibited relatively independent pattern of channel representation. In particular, channel 38 in FAST with positive seed word exhibited a relatively low correlation with channel 5 in FAST with negative seed word (*r* = −0.046, *p* > 0.05). Similarly, the valence-specific FAST channels also displayed mostly non-significant correlations with those of VFT and FAST with neutral seed word, see Table 1.

**Table 1:** Correlation matrix of depression-related task activated channels.

	VFT 8	VFT 15	VFT 16	VFT 20	FAST 5 (negative)	FAST 36 (negative)	FAST 15 (neutral)	FAST 36 (neutral)	FAST 20 (positive)	FAST 38 (positive)
VFT 8	1									
VFT 15	−0.154	1								
VFT 16	−0.088	0.577*	1							
VFT 20	0.320	−0.456	−0.372	1						
FAST 5 (negative)	0.205	−0.573*	−0.283	0.349	1					
FAST 36 (negative)	0.047	−0.829***	−0.602*	0.482	0.426	1				
FAST 15 (neutral)	−0.187	0.806***	0.644**	−0.367	−0.534*	−0.650**	1			
FAST 36 (neutral)	0.286	−0.874***	−0.438	0.541*	0.488	0.919***	−0.615*	1		
FAST 20 (positive)	0.183	−0.322	−0.085	0.706**	0.463	0.095	−0.219	0.271	1	
FAST 38 (positive)	0.166	−0.022	−0.323	0.524*	−0.046	0.169	−0.159	0.135	0.522*	1

*p < 0.05, **p < 0.01, ***p < 0.001.

Moreover, in order to clarify the efficacy of each task in identifying depression severity, multiple linear regression analyses were used to calculate the interpretability of significant channels to EPDS under each task. The results showed that each task explained 35.5% (VFT, VFT-fruits 40.9%, VFT-vegetables 31.1%, and VFT-animals 38.8%, respectively), 29.7% (FAST-positive), 28.5% (FAST-negative) and 21.2% (FAST-neural) of the EPDS variance, respectively (Table 2).

**Table 2:** Regression analysis of activated channels on the EPDS for each task.

Model	df	R^2^	Adjusted R^2^	*p*
FAST1 (negative)	19	0.361	0.285	0.022*
FAST2 (neutral)	18	0.299	0.212	0.058
FAST3 (positive)	20	0.367	0.297	0.016*
VFT	18	0.498	0.355	0.036*
VFT (fruits)	18	0.541	0.409	0.021*
VFT (vegetables)	18	0.464	0.311	0.054
VFT (animals)	18	0.524	0.388	0.026*

*p ≤ 0.05.

## 4 Discussion

The present study investigated the feasibility of the free association semantic task, a novel paradigm based on emotional seed words, as a tool for fNIRS-based assessment of perinatal depression. Our study revealed significant correlations between FAST-related oxy-Hb changes over the frontal brain regions and the Edinburgh Postnatal Depression Scale score. The correlation patterns were distinct for FAST with different seed words. The FAST-related fNIRS activities by both positive and negative seed words were substantially different from those by the neutral seed word, as well as those by the classical VFT paradigm.

The behavioral results suggested a valid execution of the FAST paradigm by the participants, as shown in Figure 4. In contrast to the VFT paradigm in which the participants were required to generate words belonged to a given category, the participants were asked to generate words only related to the concept of the preceding word in the FAST. As expected, the VFT word chains showed a consistently close distance to the target seed words, while maintaining a large and stable distance to the non-target seed words. The FAST word chains, however, showed a gradual deviation from the target seed words over time, indicating an effective execution of the task. Hereby, the FAST paradigm in the present study might offer a better reflection of the dynamic development process of individual thought flows (38, 39) as compared to VFT.

The correlation patterns between EPDS and the fNIRS activities in the FAST with positive and negative seed words could reflect a distinct mechanism as compared to the classical VFT and FAST with neutral seed word. In particular, the correlation patterns in the FAST with positive and negative seed words were predominantly observed in the fNIRS channels corresponding to the brain regions such as DLPFC and IFG; the VFT was associated with other parts such as the MTG and FPC. While the VFT findings were consistent with previous studies for executive control processing (60–62), the frontal-oriented FAST findings could imply the involvement of emotion generation and regulation (63). More specifically, the frontal part has been suggested to be related to depression (64) and previous studies using emotion-related tasks have reported depression-specific functional relevance for IFG and DLPFC (65–67). The emotion specificity of the FAST results was further supported by the observation that our FAST results with the neutral seed word were more similar to the VFT results without much frontal involvement. In addition, the correlation analyses among the channel sets of interest from different tasks (Table 1) suggested relatively independent response patterns in these tasks. Taken together, the distinct patterns observed in the FAST with emotional seed words may therefore provide a unique window into the neural underpinnings of emotional dysfunction in PD.

Furthermore, the FAST with positive and negative seed words were associated with different brain regions for representing PD severities. Specifically, as PD score increased, the positive-seed-word FAST was reflected by decreased activation of the fNIRS channels corresponding to DLPFC, whereas the negative-seed-word FAST was mainly reflected by decreased activation of the fNIRS channels corresponding to IFG. The DLPFC is linked not only to individuals’ cognitive and executive functions (68, 69), but also to the voluntary or effortful regulation of an individual’s emotional state (70, 71). Abnormally low activation of DLPFC has been widely reported in patients with depression (72, 73), which is characterized by an inability to regulate the affective processing system (74, 75). Accordingly, we speculate that individuals with a higher level of depression have a reduced capacity to mobilize positive memory experiences in free association with positive seed word, which is reflected in the weaker activation of DLPFC in neural representation. On the other hand, a negative correlation was observed between depression severities and oxy-Hb alterations in the fNIRS channels corresponding to IFG during the FAST with negative seed word. The IFG is frequently activated in response to negative emotional cues (76, 77) and plays a pivotal role in emotion recognition and evaluation, empathy, and executive function (78–81). Prior studies have revealed the role of IFG as an indicator of successful inhibition of emotional distraction, and this function was impaired in patients with depression (82, 83). Meanwhile, consistent evidence has been indicating that the IFG was hypoactive in women with subthreshold or postpartum depression, which is considered as a sensitive indicator preceding the clinical onset (84, 85). In accordance with the cognitive model of depression, deficiencies in the inhibition of negative emotional responses may engender negative biases in attention and memory, thereby resulting in symptoms such as rumination (86). Our findings align with previous studies, indicating that in the FAST induced by negative seed word, a higher depression tendency was associated with insufficient IFG activation in pregnancy. This might reflect weakened emotional inhibition control, thereby increasing the risk of individuals indulging in negative thinking during the association process. Additionally, the correlation between FAST-specific channels with positive and negative seed words was also statistically insignificant, thereby allowing complementary role of the two types of FAST for a more complete assessment of PD.

Finally, regression analyses using the fNIRS activities in each FAST session revealed significant overall regression models for FAST with the positive and the negative seed words, as well as marginally significant regression for FAST with the neutral seed word. The better model fittings for FAST with positive and negative seed words further suggest the efficacy of using FAST with emotional words. Moreover, the magnitude of R^2^ in this study is comparable to that of previous studies (45), indicating that the initial predictive efficacy of the current model is promising. Although the present study did not include fNIRS data from both positive and negative FAST, and those from VFT for a more integrated model due to the limited sample size in the present study, it is expected to see improved performance by combining these tasks together, as these tasks were associated with distinct fNIRS channels with non-significant correlations. Further studies with increased sample size are necessary to investigate how to integrate these paradigms toward more efficient and effective assessment of PD. Nevertheless, the present findings have suggested the FAST paradigm as a promising approach for PD assessment. The combination of the user-friendly nature of the FAST paradigm, characterized by its simplicity and ease of execution, with the high efficiency of being completed within tens of seconds, and the convenience of fNIRS devices for deployment in clinical settings, holds the potential to rapidly transition into clinical practice once refined (37, 87, 88).

There are also several limitations to be noted. First, the present study focused only on the late pregnancy stage. Given the sustained fluctuations in both the physical and mental states of perinatal individuals (89–91), future studies should undertake a more comprehensive assessment on participants throughout the entire perinatal process. Secondly, the behavioral data could be more extensively exploited. It could be expected that the generated word chain might be informative about the participant’s mental health state as well, especially with a larger sample size and advanced natural language processing techniques. Third, the proposed FAST paradigm could be in general applicable for depression and other types of mood disorders, but fNIRS-based studies using FAST is still limited. More extensive studies for fNIRS-based assessment using FAST is necessary for more clinical populations.

## Data Availability

The raw data supporting the conclusions of this article data will be available upon reasonable request.
