# Supplementary material for: A free association semantic task for fNIRS-based perinatal depression assessment
Source: Front Neurol. 2025 Jan 15;15:1491923. doi: 10.3389/fneur.2024.1491923 (PMC11778336; doi:10.3389/fneur.2024.1491923)
Supplement: Supplementary file 1 [file Table_1.docx]

Supplementary Material

# Supplementary Table

**Supplementary Table 1** Anatomical labels for each fNIRS channel.

| **Label of Channel** | **Brodmann Area (Talairach daemon)** | **Percentage** |
| --- | --- | --- |
| CH1 (S1-D1) | **21 - Middle Temporal Gyrus** | **0.9781** |
|  | 20 - Inferior Temporal Gyrus | 0.0219 |
| CH2 (S1-D6) | **22 - Superior Temporal Gyrus** | **0.4379** |
|  | 21 - Middle Temporal Gyrus | 0.3354 |
|  | 42 - Primary and Auditory Association Cortex | 0.2267 |
| CH3 (S2-D1) | 21 - Middle Temporal Gyrus | 0.2437 |
|  | 22 - Superior Temporal Gyrus | 0.1935 |
|  | **38 - Temporopolar area** | **0.5376** |
|  | 47 - Inferior prefrontal gyrus | 0.0251 |
| CH4 (S2-D2) | **47 - Inferior prefrontal gyrus** | **1** |
| CH5 (S2-D7) | 45 - pars triangularis Broca's area | 0.3779 |
|  | 46 - Dorsolateral prefrontal cortex | 0.0033 |
|  | **47 - Inferior prefrontal gyrus** | **0.6189** |
| CH6 (S3-D2) | **10 - Frontopolar area** | **0.6025** |
|  | 11 - Orbitofrontal area | 0.3975 |
| CH7 (S3-D3) | 10 - Frontopolar area | 0.4821 |
|  | **11 - Orbitofrontal area** | **0.5179** |
| CH8 (S3-D8) | **10 - Frontopolar area** | **1** |
| CH9 (S4-D3) | 10 - Frontopolar area | 0.3773 |
|  | **11 - Orbitofrontal area** | **0.6227** |
| CH10 (S4-D4) | 10 - Frontopolar area | 0.396 |
|  | **11 - Orbitofrontal area** | **0.4901** |
|  | 47 - Inferior prefrontal gyrus | 0.1139 |
| CH11 (S4-D9) | **10 - Frontopolar area** | **0.9831** |
|  | 11 - Orbitofrontal area | 0.0169 |
| CH12 (S5-D4) | **38 - Temporopolar area** | **0.589** |
|  | 47 - Inferior prefrontal gyrus | 0.411 |
| CH13 (S5-D5) | **21 - Middle Temporal Gyrus** | **0.9966** |
|  | 22 - Superior Temporal Gyrus | 0.0034 |
| CH14 (S5-D10) | 21 - Middle Temporal Gyrus | 0.058 |
|  | **22 - Superior Temporal gyrus** | **0.4334** |
|  | 38 - Temporopolar area | 0.2901 |
|  | 44 - pars opercularis_ part of Broca's area | 0.0307 |
|  | 45 - pars triangularis Broca's area | 0.0512 |
|  | 47 - Inferior prefrontal gyrus | 0.1365 |
| CH15 (S6-D5) | 20 - Inferior Temporal Gyrus | 0.0733 |
|  | **21 - Middle Temporal Gyrus** | **0.9267** |
| CH16 (S6-D11) | **21 - Middle Temporal Gyrus** | **0.7484** |
|  | 22 - Superior Temporal Gyrus | 0.2197 |
|  | 42 - Primary and Auditory Association Cortex | 0.0318 |
| CH17 (S7-D1) | 6 - Pre-Motor and Supplementary Motor Cortex | 0.1119 |
|  | 21 - Middle Temporal Gyrus | 0.2305 |
|  | **22 - Superior Temporal gyrus** | **0.6271** |
|  | 44 - pars opercularis_ part of Broca's area | 0.0305 |
| CH18 (S7-D6) | 4 - Primary Motor Cortex | 0.0629 |
|  | 6 - Pre-Motor and Supplementary Motor Cortex | 0.066 |
|  | 22 - Superior Temporal Gyrus | 0.2704 |
|  | 40 - Supramarginal gyrus part of Wernicke's area | 0.0063 |
|  | 42 - Primary and Auditory Association Cortex | 0.2233 |
|  | **43 - Subcentral area** | **0.3711** |
| CH19 (S7-D7) | 6 - Pre-Motor and Supplementary Motor Cortex | 0.1318 |
|  | 22 - Superior Temporal Gyrus | 0.074 |
|  | **44 - pars opercularis_ part of Broca's area** | **0.5723** |
|  | 45 - pars triangularis Broca's area | 0.2219 |
| CH20 (S7-D12) | 4 - Primary Motor Cortex | 0.0479 |
|  | **6 - Pre-Motor and Supplementary Motor cortex** | **0.7412** |
|  | 9 - Dorsolateral prefrontal cortex | 0.1693 |
|  | 43 - Subcentral area | 0.0064 |
|  | 44 - pars opercularis_ part of Broca's area | 0.016 |
|  | 45 - pars triangularis Broca's area | 0.0192 |
| CH21 (S8-D2) | **10 - Frontopolar area** | **0.4746** |
|  | 46 - Dorsolateral prefrontal cortex | 0.308 |
|  | 47 - Inferior prefrontal gyrus | 0.2174 |
| CH22 (S8-D7) | 45 - pars triangularis Broca's area | 0.2249 |
|  | **46 - Dorsolateral prefrontal cortex** | **0.7751** |
| CH23 (S8-D8) | **10 - Frontopolar area** | **0.9333** |
|  | 46 - Dorsolateral prefrontal cortex | 0.0667 |
| CH24 (S8-D13) | 9 - Dorsolateral prefrontal cortex | 0.0039 |
|  | 10 - Frontopolar area | 0.2235 |
|  | **46 - Dorsolateral prefrontal cortex** | **0.7725** |
| CH25 (S9-D3) | **10 - Frontopolar area** | **0.8746** |
|  | 11 - Orbitofrontal area | 0.1254 |
| CH26 (S9-D8) | **10 - Frontopolar area** | **1** |
| CH27 (S9-D9) | **10 - Frontopolar area** | **1** |
| CH28 (S9-D14) | 9 - Dorsolateral prefrontal cortex | 0.0036 |
|  | **10 - Frontopolar area** | **0.9964** |
| CH29 (S10-D4) | 10 - Frontopolar area | 0.2153 |
|  | 45 - pars triangularis Broca's area | 0.0255 |
|  | 46 - Dorsolateral prefrontal cortex | 0.1642 |
|  | **47 - Inferior prefrontal gyrus** | **0.5949** |
| CH30 (S10-D9) | **10 - Frontopolar area** | **0.9177** |
|  | 46 - Dorsolateral prefrontal cortex | 0.0823 |
| CH31 (S10-D10) | **45 - pars triangularis Broca's area** | **0.6595** |
|  | 46 - Dorsolateral prefrontal cortex | 0.2796 |
|  | 47 - Inferior prefrontal gyrus | 0.0609 |
| CH32 (S10-D15) | 10 - Frontopolar area | 0.1299 |
|  | **46 - Dorsolateral prefrontal cortex** | **0.8701** |
| CH33 (S11-D5) | **21 - Middle Temporal Gyrus** | **0.592** |
|  | 22 - Superior Temporal Gyrus | 0.3478 |
|  | 42 - Primary and Auditory Association Cortex | 0.0602 |
| CH34 (S11-D10) | 4 - Primary Motor Cortex | 0.0133 |
|  | 6 - Pre-Motor and Supplementary Motor cortex | 0.3123 |
|  | **22 - Superior Temporal Gyrus** | **0.5116** |
|  | 43 - Subcentral area | 0.01 |
|  | 44 - pars opercularis_ part of Broca's area | 0.1528 |
| CH35 (S11-D11) | 21 - Middle Temporal Gyrus | 0.0033 |
|  | 22 - Superior Temporal Gyrus | 0.2133 |
|  | 40 - Supramarginal gyrus part of Wernicke's area | 0.0533 |
|  | **42 - Primary and Auditory Association Cortex** | **0.67** |
|  | 43 - Subcentral area | 0.06 |
| CH36 (S11-D16) | 1 - Primary Somatosensory Cortex | 0.1429 |
|  | 2 - Primary Somatosensory Cortex | 0.0105 |
|  | 3 - Primary Somatosensory Cortex | 0.0523 |
|  | 4 - Primary Motor Cortex | 0.1254 |
|  | 6 - Pre-Motor and Supplementary Motor Cortex | 0.1986 |
|  | 22 - Superior Temporal Gyrus | 0.007 |
|  | **43 - Subcentral area** | **0.4634** |
| CH37 (S12-D7) | 9 - Dorsolateral prefrontal cortex | 0.2457 |
|  | 44 - pars opercularis_ part of Broca's area | 0.0273 |
|  | **45 - pars triangularis Broca's area** | **0.3686** |
|  | 46 - Dorsolateral prefrontal cortex | 0.3584 |
| CH38 (S12-D12) | 6 - Pre-Motor and Supplementary Motor Cortex | 0.3023 |
|  | 8 - Includes Frontal eye fields | 0.1899 |
|  | **9 - Dorsolateral prefrontal cortex** | **0.5078** |
| CH39 (S12-D13) | 8 - Includes Frontal eye fields | 0.0591 |
|  | **9 - Dorsolateral prefrontal cortex** | **0.6417** |
|  | 46 - Dorsolateral prefrontal cortex | 0.2992 |
| CH40 (S13-D8) | 9 - Dorsolateral prefrontal cortex | 0.1066 |
|  | **10 - Frontopolar area** | **0.8934** |
| CH41 (S13-D13) | 8 - Includes Frontal eye fields | 0.024 |
|  | **9 - Dorsolateral prefrontal cortex** | **0.8942** |
|  | 10 - Frontopolar area | 0.0529 |
|  | 46 - Dorsolateral prefrontal cortex | 0.0288 |
| CH42 (S13-D14) | 8 - Includes Frontal eye fields | 0.0321 |
|  | **9 - Dorsolateral prefrontal cortex** | **0.8353** |
|  | 10 - Frontopolar area | 0.1325 |
| CH43 (S14-D9) | 9 - Dorsolateral prefrontal cortex | 0.082 |
|  | **10 - Frontopolar area** | **0.918** |
| CH44 (S14-D14) | 8 - Includes Frontal eye fields | 0.0451 |
|  | **9 - Dorsolateral prefrontal cortex** | **0.8402** |
|  | 10 - Frontopolar area | 0.1148 |
| CH45 (S14-D15) | 8 - Includes Frontal eye fields | 0.0326 |
|  | **9 - Dorsolateral prefrontal cortex** | **0.8558** |
|  | 10 - Frontopolar area | 0.0558 |
|  | 46 - Dorsolateral prefrontal cortex | 0.0558 |
| CH46 (S15-D10) | 9 - Dorsolateral prefrontal cortex | 0.2937 |
|  | 44 - pars opercularis_ part of Broca's area | 0.2832 |
|  | **45 - pars triangularis Broca's area** | **0.4056** |
|  | 46 - Dorsolateral prefrontal cortex | 0.0175 |
| CH47 (S15-D15) | 8 - Includes Frontal eye fields | 0.0194 |
|  | **9 - Dorsolateral prefrontal cortex** | **0.6085** |
|  | 46 - Dorsolateral prefrontal cortex | 0.3721 |
| CH48 (S15-D16) | **6 - Pre-Motor and Supplementary Motor Cortex** | **0.8106** |
|  | 9 - Dorsolateral prefrontal cortex | 0.1894 |

*** Note:** CH: Channel, S: Source, D: Detector.
